# Psychometric validation of the sibling inventory of behavior in three- to six-year-old Chinese children

**DOI:** 10.3389/fpsyg.2023.1124518

**Published:** 2023-03-06

**Authors:** Huiyan Xu, Zhenlin Wang, Xiaozi Gao, Xiaoying Wang, Qiong Wu

**Affiliations:** ^1^Faculty of Education, Northeast Normal University, Changchun, China; ^2^Department of Psychology, The Education University of Hong Kong, Tai Po, Hong Kong SAR, China; ^3^Department of Early Childhood Education, The Education University of Hong Kong, Tai Po, Hong Kong SAR, China

**Keywords:** sibling relationship, psychometric properties, reliability, validity, measurement invariance, Chinese children

## Abstract

With increasing attention on sibling relationship studies in China, one problem that has been neglected is the limited validation of instruments used to assess these relationships. The present study evaluated the psychometric properties of the Sibling Inventory of Behavior to measure Chinese children’s sibling relationships using a stratified random sample of 590 parents of three- to six-year-olds in three economic regions. The confirmatory factor analysis obtained an adequate fit, suggesting that the Chinese version of the instrument had a six-factor structure (i.e., *Companionship*, *Empathy*, *Teaching*, *Rivalry*, *Aggression*, and *Avoidance*). It demonstrated satisfactory internal consistency as well as test–retest reliability. Results of discriminant, convergent, and criterion-related validity test also fulfilled psychometric requirements. Furthermore, the residual measurement invariance across regions was discovered. Given the importance, emergence, and tendency of sibling studies in China, having a reliable and valid instrument with robust psychometric properties is essential and impactful.

## Introduction

The relationship between siblings is influential and lasts a life time (Relva et al., 2017). Siblings act as playmates, role models, rivals, competitive partners, and objects of attachment to each other (White and Hughes, 2017). This uniquely child-driven relationship contributes significantly to children’s social, emotional, and psychological development (McHale et al., 2012; Bekkhus et al., 2016; Chen et al., 2017), especially for young children (Karavasilis Karos et al., 2007). Studies have demonstrated the influence of sibling relationships on the developmental outcomes of children in early childhood, such as prosocial behavior (Hughes et al., 2018), emotional understanding and regulation (Kramer, 2014), internalizing and externalizing symptoms (Dirks et al., 2015).

From an ecological system perspective (Bronfenbrenner, 1979), sibling relationships differ across cultures (Cicirelli, 1994; Lew-levy et al., 2020). Social and cultural values influence sibling relationship quality, which makes research that adopts a contextualized approach valuable. Sibling relationships in Asian families may differ in structure and characteristics from those in Western culture. For instance, school-aged Indonesian children reported more companionship, intimacy, and satisfaction and less conflict between siblings than American children did (French et al., 2001). The traditional “son preference” in Chinese families might contribute to parental differential treatments of sons and daughters on the part of parents, leading to poor sibling relationships in brother-and-sister dyads (Chen et al., 2017). Zhang et al. (2019) used the questionnaire for elementary school children (Furman and Buhrmester, 1985) to assess Chinese kindergartners’ sibling relationships and found that they are significantly associated with empathy, which is positively correlated with compatriot closeness, competition, and power; but negatively correlated with compatriot conflict.

After four decades of enforcing the one-child policy, starting in 2016, the Chinese government has gradually switched to policies encouraging larger family sizes with policies of universal second-child and third-child (Ren, 2022). The very recent end of one-child policy and its replacement with new policies encouraging the birth of siblings will be revolutionizing. Now a new generation of children who have siblings will prevail, and bring with it positive effects. However, research on Chinese children’s sibling relationships is unsystematic and underdeveloped, partially due to predominate single-child population in the last 40 years (Chen et al., 2017; Chen and Shi, 2017). To understand the family dynamics and child development in contemporary China under the new population policies, it is especially essential to investigate children’s sibling relationships during early childhood especially. In order to achieve such investigations, there is an urgent demand for psychometrically robust instruments assessing young children’s sibling relationships.

Meanwhile, socioeconomic factors play a role in family relationships including the sibling relationship (Skinner and McHale, 2022). Considering the various economic developmental levels across China’s vast territory, it is essential to establish measurement invariance across different economic regions. The negative relationship between fertility and economic development leads to families in underdeveloped regions tending to have more children compared with families in developed cities (Yu and Zhao, 2023). The inconsistent economic development of regions in China is also accompanied by the phenomenon of migrant and left-behind children. According to the ecological system theory (Bronfenbrenner, 1979), a family’s socioeconomic and structural status can affect interactions among family members. For instance, the family socioeconomic status effect is obvious in parent–child relationships (Ren, 2021; Huang and Wang, 2022). Therefore, an effective measure should demonstrate measurement invariance across various economic development regions.

Despite its importance and urgency, there are very few measurements of sibling relationship quality for young children (Volling and Blandon, 2005; López-Fernández et al., 2022), most of which are based on parents’ reports. The Parental Expectations and Perceptions of Children’s Sibling Relationships Questionnaire (PEPC-SRQ) describes sibling behaviors regarding sibling warmth, agonism, and rivalry/competition using 24 items (Kramer and Baron, 1995). The Sibling Relationship in Early Childhood Questionnaire (SRECQ) comprises 18 items measuring sibling positive involvement, conflict/rivalry, and avoidance (Volling et al., 2002; Chen, 2019). Schaefer and Edgerton (1981) developed the 32-item Sibling Inventory of Behavior (SIB) initially using parents’ report. It went through many modifications and has been psychometrically validated (Hetherington et al., 1992; Volling and Blandon, 2005).

What makes the SIB stand out among the available instruments is that it measures a rather comprehensive profile of sibling relationship, namely, the positive (i.e., *Companionship*, *Teaching*, and *Empathy*) and negative (i.e., *Aggression*, *Rivalry*, and *Avoidance*) interactions across six subscales. The emphasis of both detailed positive and negative interactions is in line with the argument that sibling relationships in the early years are characterized by both positive and negative behaviors (Buist and Vermande, 2014; Chen et al., 2017; White and Hughes, 2017). Especially under the specific cultural and social features of current Chinese society, young children are learning to co-live with siblings and therefore negative and positive sibling interactions co-exist in their life at present. Another advantage of the SIB lies in the good psychometric properties which were tested several times in previous studies (Schaefer and Edgerton 1981; Hetherington et al., 1992; Volling and Blandon, 2005). Reports on the SIB from different respondents, including fathers, mothers, and adolescent siblings themselves, correlate highly with each other (Volling and Blandon, 2005).

In response to the urgent need of sibling relationship measurements, Chinese researchers have developed two scales in recent years. For example, an 18-item questionnaire developed by Jiang et al. (2021) covers four subscales, namely, *Sibling interaction*, *Acceptance*, *Warmth*, and *Rivalry*. Another example is the Sibling Relationship Quality (SRQ) questionnaire for zero- to eight-year-old children, which includes three subscales of *Warmth*, *Conflict*, and *Jealousy* (Li et al., 2019; Wang et al., 2022). Both measures include positive and negative subscales. However, neither of them is as comprehensive as the SIB in coverage of a range of positive and negative subscales. Both the existing scales developed in Chinese focus on the sibling warmth in common, which is relatively generalized compared to concrete sibling interactions like keeping company, teaching, and showing empathy, etc. Studies demonstrated the significance of sibling teaching (Howe et al., 2012, 2016a,b) and empathy (Zhang et al., 2019) during the pre-school period. But there is a limited number of items about sibling teaching and empathy in SRQ (Li et al., 2019; Wang et al., 2022), and about sibling aggression in the questionnaire developed by Jiang et al. (2021). Given the fact that the SIB is widely adopted with comprehensive coverage and solid psychometric properties, a validated Chinese version of SIB (C-SIB) is warranted to advance sibling researches in our specific context.

### The current study

The main objective of the current study was to validate the C-SIB among three- to six-year-old Chinese children. Firstly, we used the forward-backward method to translate the SIB into Chinese. In the forward translation, a bilingual psychologist and a lecturer in Education translated the scale independently. Both versions were compiled, and was then back translated into English by a bilingual early childhood education graduate student. We adopted a collaborative committee approach (Douglas and Craig, 2007) to determine the accuracy and ensure the cultural appropriateness of the C-SIB. Two minor revisions were performed to meet the cultural appropriateness. The scale does not specify “child 1” as the recipient of the action as in the original SIB since families with three or more children are currently very rare in China. We changed the “child 1” into “sibling” (兄弟姐妹in Chinese). Moreover, the item of “Fusses and argues with (Child 1)” was modified to be “argues with sibling” (和兄弟姐妹吵架) because there is no appropriate expression for “fuss” in Chinese and further, “argue”(吵架) can include and replace it in meaning. Consequently, the final version of the questionnaire was administered to a larger sample size.

The validation study had three objectives. Firstly, the study would examine the factor structure of C-SIB in a Chinese sample *via* a confirmatory factor analysis. Secondly, the internal consistency, test–retest reliability, and construct and criterion validity would be examined. Finally, this study aimed to test the generalizability of the C-SIB through measurement equivalence tests across different regions representing diverse economic growth in China.

## Materials and methods

### Participants and procedure

The Institutional Review Board of Northeast Normal University reviewed and approved the study procedures and deemed them compliant with ethical standards. The study was conducted during the summer of 2022. Adopting a stratified random sampling approach, participants were recruited across three regions in China involving six provinces representing diverse economic growth as per the National Bureau of Statistics: Eastern (Hebei and Guangdong province), western and central (Shanxi, Jiangxi, and Inner Mongolia provinces), and northeastern (Liaoning province) regions (National Bureau of Statistics of China, 2011). Data were collected anonymously using a web-based survey platform to be COVID safe. In total, we approached six kindergarten principals across the three regions for recruitment. With their consent and collaboration, the survey link was sent to parents who had at least two children through online channels. Parents interested in participating in the study signed an informed consent form and responded to the survey questions online.

During the data cleanup process, we eliminated invalid responses due to missing data, short response times, or regularly repeated answers (Lee and Xu, 2003). As a result, 590 valid responses were included in the final analysis, representing 90.36% of all the parents who participated in the study. Table 1 presents the demographic data of the 590 child participants. Parents of all the participants had at least two children, one of whom was between 3 to 6 years old and currently enrolled in preschool and kindergarten. Out of the entire sample, 68 participants were invited to complete the C-SIB twice with a two-week interval for test–retest reliability.

**Table 1:** Demographic characteristics of participants (N = 590).

	*N*	%
Child gender		
Male	307	52.0
Female	283	48.0
Age in years		
3	152	25.8
4	242	41.0
5	196	33.2
Region		
Eastern	150	25.4
Northeastern	215	36.4
Western and Central	225	38.1

### Measurements

Three- to six-year-old children’s sibling relationships were assessed using the C-SIB. Parents were invited to respond to 32 items on a five-point Likert scale (1 = never, 2 = rarely, 3 = occasionally, 4 = often, and 5 = always). The questionnaire contained six subscales, including *Companionship* (6 items, e.g., Treats siblings as good friends), *Empathy* (5 items, e.g., Wants siblings to succeed), *Teaching* (4 items, e.g., Teaches siblings new skills), *Rivalry* (7 items, e.g., Tattles on siblings), *Aggression* (5 items, e.g., Teases or annoys siblings), and *Avoidance* (5 items, e.g., Stays away from sibling if possible) (Volling and Blandon, 2005). The former three subscales constitute positive sibling relationships, while the latter three make up the negative subscales. Higher scores on the positive subscales indicate better sibling relationship quality, as do lower scores on the negative subscales.

We used the Sibling Relationship Quality Questionnaire in Early Childhood (SRQ) (Li et al., 2019) to examine criterion validity of the C-SIB. The SRQ includes three subscales of *Warmth* (9 items, e.g., Helps or shares with each other), *Conflict* (4 items, e.g., Fights over toys, things, etc.), and *Jealousy* (5 items, e.g., Unpleasant or jealousy because the other one is praised). It was presented on a five-point Likert scale with good internal consistency (0.79–0.88) and test–retest reliability (0.74–0.94) (Li et al., 2019). The Cronbach’s alpha coefficients (*α*) for the three subscales in the current sample were 0.882 (*Warmth*), 0.794 (*Conflict*), and 0.871 (*Jealousy*), while the overall scale’s α was 0.785.

### Analytical plan

Data were analyzed using IBM SPSS 25.0 and M-plus 8.0. The analyzes were conducted in three steps. In the first step, we examined descriptive statistics. The confirmatory factor analysis (CFA) was then conducted with the full sample of 590 participants using a maximum likelihood (ML) estimation approach. We compared the model fits of a two-factor (i.e., positive vs. negative) structure and a six-factor (i.e., *Companionship*, *Empathy*, *Teaching*, *Rivalry*, *Aggression*, and *Avoidance*) structure (Volling and Blandon, 2005). Model fits were assessed using the chi-square test (*χ*^2^), root mean square error of approximation (RMSEA < 0.08), Tucker–Lewis index (TLI > 0.90), comparative fit index (CFI > 0.90), Akaike information criterion (AIC), Bayesian information criterion (BIC), and standardized root mean-square residual (SRMR < 0.08) (Hu and Bentler, 1999).

In the second step, we examined the subscales’ internal consistency using Cronbach’s alpha coefficient (*α*) and the composite reliability using McDonald’s omega coefficient (*ω*) (McDonald, 1999). While *α* is a prevalent measure of internal consistency, *ω* is a more accurate estimate when error covariance is present (Dunn et al., 2014). A value greater than 0.70 is considered acceptable for both *α* and *ω* (Kline, 2000; Hair et al., 2009; DeVellis, 2017). Furthermore, we calculated the temporal test–retest reliability using Pearson’s correlation coefficient.

Following the recommendations of Fornell and Larcker (1981), convergent and discriminant validity were assessed using the average variance extracted (AVE). Convergent validity is considered acceptable when AVE is larger than 0.50, indicating that more than 50% of the variance of the construct is explained by its indicators (Fornell and Larcker, 1981; Fornell and Bookstein, 1982). In addition, an AVE larger than both the maximum shared variance (MSV) and the average shared variance (ASV) indicates satisfactory discriminant validity (Hair et al., 2009; González-Rivera and Hernández-Gato, 2019). When the root of the AVE for a particular construct is greater than its correlation with all other constructs, it is also considered good discriminant validity (Fornell and Larcker, 1981). Moreover, we compared the correlations between variables in C-SIB and criterion variables in the SRQ. Specifically, good criterion-related validity is considered achieved when the positive subscales of the C-SIB correlate significantly with *Warmth*, and negative subscales correlate significantly with *Conflict* and *Jealousy* in the SRQ.

In the third step, measurement invariance was examined across the three regions of different economic growth levels. The dataset was divided into three groups, namely, eastern, northeastern, and western and central regions. A series of multi-group confirmatory factor analysis (MGCFA) were conducted with increasing invariance restrictions (configural, weak, strong, and strict invariance) (Meredith, 1993; Raju et al., 2002; Meade and Lautenschlager, 2004; Marsh et al., 2009). Cross-region measurement invariance is established if the model fit of a more restricted model is not significantly worse than the less restricted models. We focused on changes in CFI (∆CFI < 0.01) and TLI (∆TLI < 0.01) due to their robustness to sample sizes which affect *χ*^2^ difference easily (Cheung and Rensvold, 2002; Meade et al., 2008).

## Results

### Descriptive analysis

We analyzed statistical data using descriptive statistics and normality tests. As shown in Table 2, the mean scores for the items ranged from 1.48 to 4.32, with standard deviations ranging from 0.68 to 1.20. The skewness of the items ranged between −2 and + 2, while the kurtosis ranged between −7 and + 7, indicating an acceptable normal distribution (Byrne, 2010; Kim, 2013). Therefore, the ML estimation method was robust (Finney and DiStefano, 2006) and used in later CFA and measurement invariance tests.

**Table 2:** Descriptive and distribution analysis of the items (*N* = 590).

Item	*M*	SD	Skewness	Kurtosis	K-S	Item	*M*	SD	Skewness	Kurtosis	K-S
1	4.32	0.78	−1.19	1.62	0.28	17	2.52	1.06	0.32	−0.39	0.18
2	4.32	0.75	−1.07	1.37	0.28	18	2.94	1.20	0.04	−0.91	0.16
3	4.32	0.68	−0.78	0.88	0.27	19	2.47	1.16	0.37	−0.73	0.19
4	4.30	0.78	−1.24	2.04	0.27	20	2.74	1.05	0.07	−0.56	0.20
5	4.24	0.80	−0.94	0.48	0.26	21	3.02	1.15	−0.10	−0.66	0.19
6	4.09	0.89	−0.80	0.14	0.23	22	1.97	0.93	0.84	0.55	0.22
7	4.24	0.79	−0.95	0.80	0.26	23	2.16	0.96	0.50	−0.26	0.22
8	4.27	0.80	−1.10	1.28	0.27	24	2.53	0.92	0.15	−0.26	0.22
9	4.25	0.76	−0.95	1.04	0.25	25	2.82	0.92	0.03	−0.20	0.23
10	4.30	0.75	−1.02	1.26	0.27	26	1.99	0.87	0.58	−0.17	0.23
11	4.20	0.80	−0.81	0.26	0.25	27	2.03	0.96	0.70	−0.01	0.21
12	4.05	0.91	−0.87	0.44	0.25	28	1.48	0.84	1.91	3.51	0.41
13	4.00	0.92	−0.84	0.40	0.26	29	1.50	0.89	1.85	2.85	0.41
14	4.10	0.88	−0.82	0.18	0.25	30	1.51	0.95	1.86	2.56	0.43
15	3.92	0.97	−0.75	0.16	0.25	31	1.54	0.95	1.81	2.54	0.41
16	3.48	1.04	−0.36	−0.44	0.21	32	1.51	0.90	1.85	2.73	0.41

*M* = Mean; SD = Standard Deviation; K-S = Kolmogorov–Smirnov; Skewness standard error = 0.101; Kurtosis standard error = 0.201. Degrees of freedom Kolmogorov–Smirnov = 590, all the values *p* < 0.001.

### Factor structure and item discrimination

Given that the SIB has already demonstrated good psychometric properties in previous studies (Schaefer and Edgerton 1981; Hetherington et al., 1992; Volling and Blandon, 2005), we used the ML method to perform the CFA on models of the C-SIB. We have a number of variables in the analysis and our sample size was not huge (*N* = 590) especially when it was distributed into three different regions. Thus Swain’s adjustment to fit indices (Swain, 1975) was calculated using the Swain function with R (Boomsma and Herzog, 2013). Table 3 presents the results of CFA of the two- and the six-factor models. It shows that the two-factor model (i.e., *Positive* vs. *Negative*) did not fit the data well (CFI = 0.607, TLI = 0.579, RMSEA = 0.146, and SRMR = 0.14). In contrast, the six-factor model (i.e., *Companionship*, *Empathy*, *Teaching*, *Rivalry*, *Aggression*, and *Avoidance*) yielded a reasonable model fit (CFI = 0.906, TLI = 0.896, RMSEA = 0.072, and SRMR = 0.067). The Swain corrected model fitting estimates indicated slightly better model fit than the uncorrected ones.

**Table 3:** Goodness-of-fit test for analyzed models.

Model	*χ* ^2^	df	CFI (CFI_S_)	TLI (TLI_S_)	RMSEA (RMSEA_S_)	AIC	BIC	SRMR
Two-factor	6255.843	463	0.607 (0.615)	0.579 (0.588)	0.146 (0.144)	40730.471	41155.342	0.140
Six-factor	1837.645	449	0.906 (0.908)	0.896 (0.899)	0.072 (0.071)	36340.272	36826.466	0.067
Six-factor + 3 MI	1333.618	447	0.940 (0.942)	0.933 (0.935)	0.058 (0.057)	35840.245	36335.199	0.059

*χ*^2^ = Chi-square test; CFI = comparative fit index; CFI_S_ = Swain-corrected CFI；TLI = Tucker–Lewis Index; TLI_S_ = Swain-corrected Tucker–Lewis Index; RMSEA = root mean square error of approximation; RMSEA_S_ = Swain-corrected root mean square error of approximation; AIC = Akaike Information Criterion; BIC = Bayesian Information Criterion; SRMR = standardized root meansquare residual; MI = Modified Indicies; All statistics *χ*^2^ are significant, *p* < 0.001.

A closer examination of modification indices (MI) of the six-factor model suggested that the model fit could be improved by loading item 22 (“Resents sibling”) onto the factor of *Aggression* (MI = 113.255) instead of *Rivalry*, and by freeing the covariance between Item 3 (“Has fun at home with siblings”) and Item 4 (“Treats siblings as good friends”) in the subscale of *Companionship*; and between Item 7 (“Is pleased by progress siblings make”) and Item 8 (“Wants siblings to succeed”) in *Empathy*. The model fit indexes of the final six-factor C-SIB with MIs were acceptable, with *χ*^2^ = 1333.618, df = 447, *p* < 0.001, and CFI = 0.940, TLI = 0.933, RMSEA = 0.058, and SRMR = 0.059, suggesting a viable six-factor model (Hu and Bentler, 1995). The correlation coefficients between the positive and negative subscales in the six-factor model were relatively small or negative in the current study (Table 4), suggesting that a second-order or higher-order model is unlikely.

**Table 4:** Convergent, discriminant, and criterion validity.

	AVE (Root)	MSV	ASV	Warmth	Conflict	Jealousy	1	2	3	4	5	6
1. Com.	0.62 (0.79)	0.56	0.22	0.752***	−0.178***	−0.159***	-					
2. Emp.	0.69 (0.83)	0.56	0.25	0.733***	−0.257***	−0.196***	0.736***	-				
3. Tch.	0.73 (0.85)	0.56	0.21	0.683***	−0.162***	−0.097*	0.653***	0.745***	-			
4. Riv.	0.54 (0.73)	0.41	0.10	0.044	0.536***	0.470***	0.097*	0.080	0.127**	-		
5. Agg.	0.54 (0.73)	0.41	0.12	−0.219***	0.725***	0.554***	−0.165***	−0.252***	−0.154***	0.642***	-	
6. Avo.	0.91 (0.95)	0.10	0.06	−0.293***	0.287***	0.337***	−0.310***	−0.267***	−0.197***	0.124**	0.321***	-

AVE = average variance extracted; MSV = maximum shared variance; ASV = average shared variance; Com. = Companionship; Emp. = Empathy; Tch. = Teaching; Riv. = Rivalry; Agg. = Aggression; Avo. = Avoidance. *** = significant correlations *p* < 0.001; ** = significant correlations *p* < 0.01; * = significant correlations *p* < 0.05.

The items’ factor loadings are shown in Table 5. The items loaded on their respective factors with loading values ranging from 0.636 to 0.925. Furthermore, we used item-total correlations (rbis) to demonstrate the discrimination indices of the six factors. The rbis indices of all items were greater than 0.30 (0.556–0.924, *p*s < 0.001), suggesting that all items obtained discrimination indices (Kline, 2005).

**Table 5:** Item-total correlation index and CFA factor loadings.

Item	rbis	CFA factor loading	Item	rbis	CFA factor loading
1	0.788	0.713	17	0.775	0.727
2	0.857	0.819	18	0.816	0.743
3	0.820	0.744	19	0.784	0.728
4	0.863	0.794	20	0.829	0.819
5	0.808	0.795	21	0.783	0.741
6	0.851	0.837	22	0.777	0.744
7	0.870	0.766	23	0.800	0.771
8	0.870	0.764	24	0.824	0.800
9	0.866	0.864	25	0.762	0.703
10	0.907	0.916	26	0.781	0.710
11	0.853	0.839	27	0.753	0.656
12	0.887	0.846	28	0.586	0.852
13	0.924	0.923	29	0.574	0.875
14	0.893	0.865	30	0.556	0.915
15	0.869	0.786	31	0.577	0.925
16	0.710	0.636	32	0.556	0.919

rbis = item-total correlation index.

### Reliability

To confirm the reliability of the questionnaire, we conducted reliability tests which included Cronbach’s internal consistency, composite and test–retest reliabilities. As shown in Table 6, both the Cronbach’s alphas (0.873–0.954) and composite reliabilities (0.874–0.954) for the six factors exceed 0.70, indicating good internal reliability. In addition, the Pearson correlation coefficients between test and retest scores ranged from 0.641 to 0.766, suggesting acceptable test–retest reliability (Koo and Li, 2016).

**Table 6:** Means, standard deviation, and reliability tests of subscales.

	*M*	SD	*α*	*ω*	Test–retest
Com.	25.59	3.89	0.909	0.906	0.747***
Emp.	21.27	3.41	0.921	0.918	0.649***
Tch.	16.06	3.29	0.915	0.916	0.651***
Riv.	17.16	5.22	0.873	0.875	0.641***
Agg.	13.50	4.35	0.873	0.874	0.761***
Avo.	6.14	2.53	0.954	0.954	0.766***

*M* = Mean; SD = standard deviation; α = Cronbach’s alpha coefficient; w = omega coefficient; Com. = Companionship; Emp. = Empathy; Tch. = Teaching; Riv. = Rivalry; Agg. = Aggression; Avo. = Avoidance; *** = significant correlations *p* < 0.001.

### Convergent and discriminant validity

We performed convergent and discriminant validity analysis using the index of AVE. The AVEs ranged from 0.54 to 0.91 (see Table 4), indicating acceptable convergent validity. They were larger than the respective MSVs (0.10–0.56) and ASVs (0.06–0.25) as well. The roots of AVEs (0.73–0.95) were greater than respective intercorrelations with other subscales, suggesting good discriminant validity. The positive factors moderately correlated with each other (0.653–0.745, *p*s < 0.001), as did the negative factors (0.321–0.642, *p*s < 0.001), except for that between the factor of *Rivalry* and *Avoidance*, which demonstrated a small effect sized correlation (0.124, *p* < 0.01).

### Criterion validity

The concurrent criterion validity with the SRQ is demonstrated in Table 4. The positive C-SIB factors (i.e., *Companionship*, *Empathy*, *Teaching*) correlated moderately with the *Warmth* in the SRQ, with the *r* ranging between 0.683 and 0.752 (*p*s < 0.001). The negative factors of C-SIB (i.e., *Rivalry*, *Aggression*, and *Avoidance*) correlated with the *Conflict* and *Jealousy* in the SRQ, with small to moderate effect sizes ranging from 0.287 to 0.725 (*p*s < 0.001).

### Cross-region measurement invariance

Stepwise multi-group CFAs were used to examine the measurement invariance in order to compare constructs of the questionnaire across three economic regions. The Measurement invariance was assessed by fitting a sequence of models, where with every model a new set of model parameters was set to be equal across groups (Van De Schoot et al., 2012). Table 7 shows that all configural, metric, scalar, and residual invariance models yielded an acceptable model fit, which means the same factor structure fitted the sibling relationship across regions.

**Table 7:** Invariance Analysis across regions.

Model	*χ* ^2^	df	CFI	TLI	AIC	SRMR	RMSEA (90%CI)	∆*χ*^2^(∆df)	∆CFI	∆TLI
M1-Configural	2849.42	1,353	0.901	0.891	36276.701	0.069	0.075 (0.071,0.079)			
M2-Metric	2912.738	1,401	0.899	0.893	36244.019	0.073	0.074 (0.070,0.078)	63.318 (48)	−0.002	0.002
M3-Scalar	2970.182	1,451	0.899	0.896	36201.463	0.074	0.073 (0.069,0.077)	57.444 (50)	0	0.003
M4-Residual	3133.129	1,515	0.892	0.894	36236.409	0.077	0.074 (0.070,0.077)	162.947 (64) **	−0.007	−0.002

*χ*^2^ = Chi-square test; CFI = comparative fit index; TLI = Tucker–Lewis Index; AIC = Akaike Information Criterion; SRMR = standardized root meansquare residual; RMSEA = root mean square error of approximation; All statistics *χ*^2^ are significant, *p* < 0.001. ** = significant correlations *p* < 0.01.

To test configural invariance, we fit the model 1 that was specified onto each of the region groups, leaving all factor loadings and item intercepts free to vary for each group. Results demonstrated a good multi-group model fit (*χ*^2^ = 2849.42, *df* = 1,353, *p* < 0.001, and CFI = 0.901, TLI = 0.891, RMSEA = 0.075, and SRMR = 0.069), suggesting that the overall factor structure holds up similarly for all groups. The factor model can be applied to all groups in general, which is a starting point. More importantly, we then compared four nested models with increasing restrictions on parameters based on the changes of CFIs and TLIs. Firstly, we constrained the factor loadings to be equivalent across groups and tested metric invariance by contrasting Model l with Model 2. The increase in *χ*^2^ was not statistically significant (∆*χ*^2^ = 63.318, *p* > 0.05), and the ∆CFI (−0.002) and ∆TLI (0.002) were smaller than 0.01, suggesting that the factor loadings were invariant across the three regions. Secondly, we constrained the item intercepts to be equivalent across groups and compared Model 2 with Model 3. Results revealed that the *χ^2^* change was not significant (∆*χ*^2^ = 57.444, *p* > 0.05) and the ∆CFI (0.000) and ∆TLI (0.003) were smaller than 0.01, suggesting item intercepts were invariant across regions. The full scalar measurement invariance was supported too. Finally, we compared Model 3 with Model 4 to examine whether item residuals were different across the three regions. The result showed that both the ∆CFI (−0.007) and ∆TLI (−0.002) were less than 0.01, supporting full residual invariance. The test of difference in *χ*^2^ was statistically significant (∆*χ*^2^ = 162.947, *p* < 0.01), likely due to the large sample size. It leads to the situation where measurement parameters are identical across different groups, thereby enforcing zero tolerance for deviations (Van De Schoot et al., 2015). To conclude, the residual invariance (invariance of factor structure, item loadings, item intercepts and residual) exists across three regions and this confirms the measurement invariance of the C-SIB.

## Discussion

The sibling relationship is essential in the lives of young children. A psychometrically valid assessment of sibling relationship lays the foundation for future empirical research on this topic. Cross-cultural studies on sibling relationships are scarce because of methodological limitations, such as the absence of a universal measurement (White and Hughes, 2017). Thus, the emerging sibling relationship research in China requires validation of reliable and valid instruments. The main contribution of the present study was to provide a psychometrically validated measure of C-SIB in a large representative sample of three- to six-year-old Chinese children timely. Validating the SIB, which evaluates both the positive and negative aspects of sibling relationships with comprehensive information, is crucial. The final C-SIB with 32 items has appropriate psychometric properties to measure the quality of sibling relationships among young Chinese children across a diverse range of economic developmental regions.

The C-SIB measures a rather comprehensive profile of young children’s sibling relationship, including both the positive and negative aspects. The scores on the positive subscales correlated highly with each other. Among the negative subscales, *Aggression* correlated highly with *Rivalry* and moderately with *Avoidance*. However, the correlation between *Avoidance* and *Rivalry* was small, which is reasonable considering sibling rivalry implies confrontation instead of avoidance. Another possible reason for the low correlation between *Rivalry* and *Avoidance* might be associated with the children’s age in this sample. We did not ask parents to specify whether the target preschool-aged children had a younger sibling or an older sibling. However, factoring in the very recent policy change, the majority of preschool-aged children should have a younger sibling, and avoidance between very young siblings should be less prevalent. This speculation was confirmed by the low mean score of *Avoidance* (Table 6). However, the correlations between the positive subscales and the negative ones were either trivial or negative, confirming the distinctive valences between them. The positive subscales and the negative ones address rather different aspects of sibling relationship rather than the same constructs that represent the two extremes on the same continuum. Having multiple indicators across both positive and negative subscales provides a more comprehensive understanding of the complex sibling relationship.

Even within the positive subscales, the SIB and the current validated C-SIB include more dimensions than other measures. Similar to other studies on sibling relationship (Volling et al., 2002; Chen, 2019), the negative subscales of SIB and C-SIB also cover a comprehensive range of aspects including *Rivalry*, *Aggression*, and *Avoidance*. In contrast, previous instruments developed in China include *Warmth* (Li et al., 2019; Jiang et al., 2021; Wang et al., 2022) and *Acceptance* Jiang et al., 2021 as the only indicators of positive sibling relationships. In the SIB and C-SIB, however, positive sibling interactions are composed of the more comprehensive three subscales of *Companionship*, *Empathy*, and *Teaching*. Howe et al. (2012, 2016a,b) observed sibling interactions and found that sibling teaching is common during the period of early childhood, which provides a rich context for young children’s early learning and development. Furthermore, empathy and kindness are important aspects in positive sibling interactions (Barata et al., 2022) as well as in Chinese kindergartener siblings’ activities (Zhang et al., 2019).

Probably associated with the one-child policy, for generations in which it was uncommon to have siblings; having a sibling brought with it a few negative connotations, thus influencing the relationships between siblings (in particular between sons and daughters). For example, extreme conflicts between siblings (e.g., the first child killing the second child, or the first child verbally abusing the second child) have become commonplace in recent years (Chen et al., 2017). Now, a new generation of children for whom having siblings will be more commonplace and who will live their condition positively is emerging. Therefore detailing positive sibling interactions, such as keeping company, learning from each other, and empathy, will emerge. The comprehensive content of positive sibling interactions in the validated C-SIB is valuable.

The presence of measurement invariance suggests that the six-factor, 32-item structure of the C-SIB was invariant across different economic regions. China is a vast country with the largest population in the world and the regional economic differences are huge (Chen and Luo, 2011). From an ecological system perspective (Bronfenbrenner, 1979), family socioeconomic background influences parental marital satisfaction and its association with positive and negative sibling relationships (Skinner and McHale, 2022). It is important for a measure to demonstrate measurement invariance across different regions of economic development. The stratified randomized sampling of the current study enables a representative sample. The findings provided strong support for the cross-region invariance in this study. Respondents from different economic regions interpret the C-SIB in a conceptually similar way. Therefore, it is a robust measure that can be applied in sibling relationship studies in the mainland China, regardless of regions.

## Conclusion

Based on fitting indices and multifaceted information (Kline, 2010), the present study concluded that the 32-item C-SIB has appropriate and promising psychometric properties. This implies adequate reliability and a solid internal structure of six latent factors, namely, *Companionship*, *Empathy*, *Teaching*, *Rivalry*, *Aggression*, and *Avoidance*. With the emerging generation of children growing up with siblings post the one-child era, the current study contributes a much needed psychometrically reliable and valid measure for Chinese children’s sibling relationship during early childhood. The C-SIB provides a comprehensive coverage and measures both positive and negative sibling relationships. Most importantly, the generalizability of the C-SIB in different economic regions has practical implications for future researches in China.

## Limitations and future directions

The interpretation of the current results should be done with caution due to several limitations. First, the lack of family background and socioeconomic status in the data makes it difficult to discern the potential impact of familial and parental factors in sibling relationships. Parents’ educational background, marital status and social support system, as well as siblings’ age, gender composition, and developmental issues could affect sibling relationships. Whether it was the mother or father reporting on the survey might affect the results too. Future research adopting the C-SIB should consider the familial and parental rearing factors as well as children’s development issues. Second, more evidence is needed to assess the appropriateness of the questionnaire for use with children of other age groups. Thus we can take it a step further to verify its advantages and superiority compared to other measurements. This calls for more validation studies across different age groups of children in China in the future. Third, we only used the SRQ developed by Li et al. (2019) as the criterion-related validity scale in this study because of its ready availability. In the future, multiple scales can be considered for standard validity verification.

## Data availability statement

The raw data supporting the conclusions of this article will be made available by the authors, without undue reservation.

## Ethics statement

The studies involving human participants were reviewed and approved by Institutional Review Board of Northeast Normal University. Written informed consent to participate in this study was provided by the participants’ legal guardian/next of kin.

## Author contributions

HX, ZW, and XW designed the study and devised the project. HX and ZW took the lead in writing the manuscript. XW was responsible for project supervision. XG and QW verified the analytical methods and contributed to manuscript revision. All authors contributed to the article and approved the submitted version.

## Funding

This study was supported by a grant of the National Social Science Foundation of China: “Path and Strategy of Sustainable Early Childhood Education Development in China” (Reference No.: AHA200010). This study was also partially funded by a research grant (RG 75/20-21R) awarded to ZW by The Education University of Hong Kong.

## Conflict of interest

The authors declare that the research was conducted in the absence of any commercial or financial relationships that could be construed as a potential conflict of interest.

## Publisher’s note

All claims expressed in this article are solely those of the authors and do not necessarily represent those of their affiliated organizations, or those of the publisher, the editors and the reviewers. Any product that may be evaluated in this article, or claim that may be made by its manufacturer, is not guaranteed or endorsed by the publisher.

## References

[ref1] Barata Ö. Acar I. H. Bostancı S. (2022). Associations among adolescents’ mindfulness, sympathy, cognitive empathy, and sibling relationships. Psychol. Rep. 2022:1097951. doi: 10.1177/00332941221097951, PMID: 35491664

[ref2] Bekkhus M. Brendgen M. Czajkowski N. O. Vitaro F. Dionne G. Boivin M. (2016). Associations between sibling relationship quality and friendship quality in early adolescence: looking at the case of twins. Twin Res. Hum. Genet. 19, 125–135. doi: 10.1017/thg.2016.9, PMID: 26952576

[ref3] Boomsma A. Herzog W. (2013). *R Function Swain: Correcting Structural Equation Model Tstatistics and Indexes Under Small-Sample and/or Large-Model Conditions*. Groningen: University of Groningen.

[ref4] Bronfenbrenner U. (1979). The Ecology of Human Development: Experiments by Nature and Design. Cambridge: Harvard University Press.

[ref5] Buist K. L. Vermande M. (2014). Sibling relationship patterns and their associations with child competence and problem behavior. J. Fam. Psychol. 28, 529–537. doi: 10.1037/a0036990, PMID: 24866727

[ref6] Byrne B. M. (2010). Structural Equation Modeling with AMOS: Basic Concepts, Applications, and Programming. Abingdon: Routledge.

[ref7] Chen B. (2019). Chinese mothers’ sibling status, perceived supportive co-parenting, and their children’s sibling relationships. J. Child Fam. Stud. 28, 684–692. doi: 10.1007/s10826-018-01322-3

[ref8] Chen R. Luo H. (2011). Analysis of the compartmentalization of the standard economic area in China. Econ. Res. Guid. 8, 279–281.

[ref9] Chen B. Shi Z. (2017). Parenting in families with two children. Adv. Psychol. Sci. 25, 1172–1181. doi: 10.3724/SP.J.1042.2017.01172

[ref10] Chen B. Zhao Y. Han W. Wang Y. Wu J. Yue X.. (2017). Sibling relationships: forms, causes and consequences. Adv. Psychol. Sci. 25, 2168–2178. doi: 10.3724/SP.J.1042.2017.02168

[ref11] Cheung G. W. Rensvold R. B. (2002). Evaluating goodness-of-fit indexes for testing measurement invariance. Struct. Equ. Model. 9, 233–255. doi: 10.1207/S15328007SEM0902_5

[ref12] Cicirelli V. G. (1994). Sibling relationships in cross-cultural perspective. J. Marriage Fam. 56, 7–20. doi: 10.2307/352697

[ref13] DeVellis R. F. (2017). Scale Development: Theory and Applications. Thousand Oaks, CA: Sage.

[ref14] Dirks M. A. Persram R. Recchia H. E. Howe N. (2015). Sibling relationships as sources of risk and resilience in the development and maintenance of internalizing and externalizing problems during childhood and adolescence. Clin. Psychol. Rev. 42, 145–155. doi: 10.1016/j.cpr.2015.07.003, PMID: 26254557

[ref15] Douglas S. P. Craig C. S. (2007). Collaborative and iterative translation: an alternative approach to back translation. J. Int. Mark. 15, 30–43. doi: 10.1509/jimk.15.1.030

[ref16] Dunn T. J. Baguley T. Brunsden V. (2014). From alpha to omega: a practical solution to the pervasive problem of internal consistency estimation. Br. J. Psychol. 105, 399–412. doi: 10.1111/bjop.12046, PMID: 24844115

[ref17] Finney S. J. DiStefano C. (2006). “Non-normal and categorical data in structural equation models” in A Second Course in Structural Equation Modeling. eds. Hancock G. R. Mueller R. O. (Greenwich, CT: Information Age), 269–314.

[ref18] Fornell C. Bookstein F. L. (1982). Two structural equation models: LISREL and PLS applied to consumer exit-voice theory. J. Mark. Res. 19, 440–452. doi: 10.1177/002224378201900406

[ref19] Fornell C. Larcker D. F. (1981). Evaluating structural equation models with unobservable variables and measurement error. J. Mark. Res. 18, 39–50. doi: 10.1177/002224378101800104

[ref20] French D. C. Rianasari M. Pidada S. Nelwan P. Buhrmester D. (2001). Social support of Indonesian and U.S. children and adolescents by family members and friends. Merrill Palmer Q. 47, 377–394. doi: 10.1353/mpq.2001.0015

[ref21] Furman W. Buhrmester D. (1985). Children’s perceptions of the qualities of sibling relationships. Child Dev. 56, 448–461. doi: 10.2307/1129733 3987418

[ref22] González-Rivera J. A. Hernández-Gato I. (2019). Conflicts in romantic relationships over facebook use: validation and psychometric study. Behav. Sci. 9:18. doi: 10.3390/bs9020018, PMID: 30744152 PMC6406987

[ref23] Hair J. F. Black W. C. Babin B. J. Anderson R. E. (2009). Multivariate Data Analysis 7th. London, United Kingdom: Pearson.

[ref24] Hetherington E. M. Clingempeel W. G. Anderson E. R. Deal J. E. Hagan M. S. Hollier E. A.. (1992). Coping with marital transitions: a family systems perspective. Monogr. Soc. Res. Child Dev. 57, i–238. doi: 10.2307/1166050

[ref25] Howe N. Adrien E. Della Porta S. Peccia S. Recchia H. Osana H. P.. (2016a). ‘Infinity means it goes on forever’: Siblings’ informal teaching of mathematics. Infant Child Dev. 25, 137–157. doi: 10.1002/icd.1928

[ref26] Howe N. Della Porta S. Recchia H. Ross H. (2016b). “Because if you don’t put the top on, it will spill”: a longitudinal study of sibling teaching in early childhood. Dev. Psychol. 52, 1832–1842. doi: 10.1037/dev0000193 27710000

[ref27] Howe N. Recchia H. Porta S. D. Funamoto A. (2012). “The driver doesn’t sit, he stands up like the Flintstones!”: sibling teaching during teacher-directed and self-guided tasks. J. Cogn. Dev. 13, 208–231. doi: 10.1080/15248372.2011.577703

[ref28] Hu L. T. Bentler P. M. (1995). “Evaluating model fit” in Structural Equation Modeling: Issues, Concepts, and Applications. ed. Hoyle R. (Thousand Oaks, CA: Sage), 76–99.

[ref29] Hu L. T. Bentler P. M. (1999). Cutoff criteria for fit indexes in covariance structure analysis: conventional criteria versus new alternatives. Struct. Equ. Model. Multidiscip. J. 6, 1–55. doi: 10.1080/10705519909540118

[ref30] Huang H. Wang X. (2022). The effect of family socioeconomic status on migrant preschool children’s problem behaviors: the chain mediating role of family resilience and child–parent relationship. J. Psychol. Sci. 45, 315–322. doi: 10.16719/j.cnki.1671-6981.20220207

[ref31] Hughes C. McHarg G. White N. (2018). Sibling influences on prosocial behavior. Curr. Opin. Psychol. 20, 96–101. doi: 10.1016/j.copsyc.2017.08.015, PMID: 28858773

[ref32] Jiang M. Cao X. Huang Q. Wu S. Chen X. (2021). Exploring the structure of sibling relationships among preschool children in China and developing a questionnaire. Front. Psychol. 12:745165. doi: 10.3389/fpsyg.2021.745165, PMID: 35111096 PMC8801529

[ref33] Karavasilis Karos L. Howe N. Aquan-Assee J. (2007). Reciprocal and complementary sibling interactions, relationship quality and socio-emotional problem solving. Infant Child Dev. 16, 577–596. doi: 10.1002/icd.492

[ref34] Kim H.-Y. (2013). Statistical notes for clinical researchers: assessing normal distribution (2) using skewness and kurtosis. Restor. Dent. Endod. 38, 52–54. doi: 10.5395/rde.2013.38.1.52, PMID: 23495371 PMC3591587

[ref35] Kline P. (2000). The Handbook of Psychometric Testing. Abingdon: Routledge.

[ref36] Kline T. J. (2005). Psychological Testing: A Practical Approach to Design and Evaluation. Thousand Oaks, CA: Sage.

[ref37] Kline R. B. (2010). Principles and Practice of Structural Equation Modeling. New York: The Guilford Press.

[ref38] Koo T. K. Li M. Y. (2016). A guideline of selecting and reporting intraclass correlation coefficients for reliability research. J. Chiropr. Med. 15, 155–163. doi: 10.1016/j.jcm.2016.02.012, PMID: 27330520 PMC4913118

[ref39] Kramer L. (2014). Learning emotional understanding and emotion regulation through sibling interaction. Early Educ. Dev. 25, 160–184. doi: 10.1080/10409289.2014.838824

[ref40] Kramer L. Baron L. A. (1995). Parental perceptions of children’s sibling relationships. Fam. Relat. 44, 95–103. doi: 10.2307/584746

[ref41] Lee S. Y. Xu L. (2003). Case-deletion diagnostics for factor analysis models with continuous and ordinal categorical data. Soc. Methods Res. 31, 389–419. doi: 10.1177/0049124102239081

[ref42] Lew-Levy S. Kissler S. M. Boyette A. H. Crittenden A. N. Mabulla I. A. Hewlett B. S. (2020). Who teaches children to forage? Exploring the primacy of child-to-child teaching among Hadza and BaYaka hunter-gatherers of Tanzania and Congo. Evol. Hum. Behav. 41, 12–22. doi: 10.1016/j.evolhumbehav.2019.07.003

[ref43] Li Y. Liu T. Zhao J. Li Y. Niu X. (2019). Development of parental perceptions of the questionnaire of children’s sibling relationship quality in early childhood. Res. Mod. Basic Educ. 36, 166–174.

[ref44] López-Fernández G. Gómez-Benito J. Kramer L. Barrios M. (2022). Spanish validation of the parental expectations and perceptions of Children’s sibling relationships questionnaire. Fam. Relat. 2022:12699. doi: 10.1111/fare.12699

[ref45] Marsh H. W. Muthén B. Asparouhov T. Lüdtke O. Robitzsch A. Morin A. J.. (2009). Exploratory structural equation modeling, integrating CFA and EFA: application to students’ evaluations of university teaching. Struct. Equ. Model. Multidiscip. J. 16, 439–476. doi: 10.1080/10705510903008220

[ref46] McDonald R. P. (1999). Test Theory: A Unified Treatment. Mahwah: Lawrence Erlbaum.

[ref47] McHale S. M. Updegraff K. A. Whiteman S. D. (2012). Sibling relationships and influences in childhood and adolescence. J. Marriage Fam. 74, 913–930. doi: 10.1111/j.1741-3737.2012.01011.x, PMID: 24653527 PMC3956653

[ref48] Meade A. W. Johnson E. C. Braddy P. W. (2008). Power and sensitivity of alternative fit indices in tests of measurement invariance. J. Appl. Psychol. 93, 568–592. doi: 10.1037/0021-9010.93.3.568, PMID: 18457487

[ref49] Meade A. W. Lautenschlager G. J. (2004). A comparison of item response theory and confirmatory factor analytic methodologies for establishing measurement equivalence/invariance. Organ. Res. Methods 7, 361–388. doi: 10.1177/1094428104268027

[ref50] Meredith W. (1993). Measurement invariance, factor analysis and factorial invariance. Psychometrika 58, 525–543. doi: 10.1007/bf02294825

[ref51] National Bureau of Statistics of China. (2011). *The Division Method of Eastern, Western, Central and Northeastern Regions. Towards Standardized and Unified China Statistics*. Available at: http://www.stats.gov.cn/ztjc/zthd/sjtjr/dejtjkfr/tjkp/201106/t20110613_71947.htm

[ref52] Raju N. S. Laffitte L. J. Byrne B. M. (2002). Measurement equivalence: a comparison of methods based on confirmatory factor analysis and item response theory. J. Appl. Psychol. 87, 517–529. doi: 10.1037/0021-9010.87.3.517, PMID: 12090609

[ref53] Relva I. C. Fernandes O. M. Alarcao M. Graham-Bermann S. Lopes P. (2017). Psychometric proprieties and construct validity of the brother–sister questionnaire in a sample of Portuguese adolescents. J. Fam. Violence 32, 333–340. doi: 10.1007/s10896-016-9851-x

[ref54] Ren C. (2021). Family socioeconomic status effect: the serial mediating roles of family–school, parent–child and teacher–student relationship. J. Sch. Stud. 18, 72–81.

[ref55] Ren Y. (2022). Theoretical and practical issues on continuous reforms of China’s fertility policy. J. Guangzhou Univ. 21, 91–104.

[ref56] Schaefer E. S. Edgerton M. (1981). The Sibling Inventory of Behavior. Chapel Hill: University of North Carolina.

[ref57] Skinner O. D. McHale S. M. (2022). Context matters: longitudinal associations between marital relationships and sibling relationships in Black families. Fam. Relat. 71, 987–1003. doi: 10.1111/fare.12658

[ref58] Swain A. J. (1975). *Analysis of Parametric Structures for Variance Matrices. Unpublished Doctoral Dissertation*. Adelaide SA: University of Adelaide.

[ref59] Van De Schoot R. Lugtig P. Hox J. (2012). A checklist for testing measurement invariance. Eur. J. Dev. Psychol. 9, 486–492. doi: 10.1080/17405629.2012.686740

[ref60] Van De Schoot R. Schmidt P. De Beuckelaer A. Lek K. Zondervan-Zwijnenburg M. (2015). Editorial: Measurement Invariance. Front. Psychol. 6:1064. doi: 10.3389/fpsyg.2015.01064, PMID: 26283995 PMC4516821

[ref61] Volling B. L. Blandon A. Y. (2005). “Positive indicators of sibling relationship quality: the sibling inventory of behavior” in What do Children need to Flourish? (Berlin: Springer), 203–219.

[ref62] Volling B. L. McElwain N. L. Miller A. L. (2002). Emotion regulation in context: the jealousy complex between young siblings and its relations with child and family characteristics. Child Dev. 73, 581–600. doi: 10.1111/1467-8624.00425, PMID: 11949910

[ref63] Wang Y. Z. Li Y. Liu T. T. Zhao J. J. Li Y. J. Niu X. B. (2022). Understanding relationships within cultural contexts: developing an early childhood sibling relationship questionnaire in China. Fam. Relat. 71, 220–237. doi: 10.1111/fare.12586

[ref64] White N. Hughes C. (2017). Why Siblings Matter: The Role of Brother and Sister Relationships in Development and Well-being. Abingdon: Routledge.

[ref65] Yu S. Zhao P. (2023). Fertility rate and eonomic development–an empirical study based on quantile regression. J. Hunan Univ. 37, 58–65. doi: 10.16339/j.cnki.hdxbskb.2023.01.009

[ref66] Zhang R. Z. Cao X. J. Ran G. M. Xia Y. C. (2019). The effect of sibling relationship quality on. First-born young children’s empathy. Stud. Early Child. Educ. 8, 52–63. doi: 10.13861/j.cnki.sece.2019.08.005

